# The Genotype Specific Competitive Ability Does Not Correlate with Infection in Natural *Daphnia magna* Populations

**DOI:** 10.1371/journal.pone.0001280

**Published:** 2007-12-05

**Authors:** Florian Altermatt, Dieter Ebert

**Affiliations:** 1 Zoologisches Institut, Universität Basel, Basel, Switzerland; 2 Tvärminne Zoological Station, Hanko, Finland; Lund University, Sweden

## Abstract

**Background:**

Different evolutionary hypotheses predict a correlation between the fitness of a genotype in the absence of infection and the likelihood to become infected. The cost of resistance hypothesis predicts that resistant genotypes pay a cost of being resistant and are less fit in the absence of parasites. The inbreeding-infection hypothesis predicts that the susceptible individuals are less fit due to inbreeding depression.

**Methods and Results:**

Here we tested if a host's natural infection status was associated with its fitness. First, we experimentally confirmed that cured but formerly infected *Daphnia magna* are genetically more susceptible to reinfections with *Octosporea bayeri* than naturally uninfected *D. magna*. We then collected from each of 22 populations both uninfected and infected *D. magna* genotypes. All were treated against parasites and kept in their asexual phase. We estimated their relative fitness in an experiment against a tester genotype and in another experiment in direct competition. Consistently, we found no difference in competitive abilities between uninfected and cured but formerly infected genotypes. This was the case both in the presence as well as in the absence of sympatric parasites during the competition trials.

**Conclusions:**

Our data do not support the inbreeding-infection hypothesis. They also do not support a cost of resistance, however ignoring other parasite strains or parasite species. We suggest as a possible explanation for our results that resistance genes might segregate largely independently of other fitness associated genes in this system.

## Introduction

Parasites and parasitoids are ubiquitous in nature [1]–[4]. They harm the infected hosts to various degrees and thereby impose selection. Usually only a subset of individuals within a host population is infected [5]–[7], either due to genetic or due to epidemiological reasons. For example, hosts of one sex, of a certain age, in certain phenotypic conditions, with a higher inbreeding level or of specific genotypes may be more often found to be infected by parasites [8]–[11]. Furthermore, genetic variation for parasite infectivity and virulence can be found among parasites [12], among hosts [13], [14] or as an interaction of both [15]. As a consequence of this, hosts may evolve defenses to resist parasites [16], [17] and these might correlate with other host fitness components. Different evolutionary hypotheses predict a correlation between a genotype's susceptibility to infection and other fitness components. Due to the evolutionary and ecological significance of parasites, such correlations are of general interest.

The cost of resistance hypothesis predicts that resistant hosts pay a cost for being resistant in the absence of the parasite [16], [18]. In the presence of parasites, resistant genotypes profit from their resistance and are expected to be good competitors, while the susceptible genotypes are more likely to get infected [5], [6], and are therefore worse competitors. Contrary, in the absence of parasites hosts carrying resistance genes would have a lower competitive ability than susceptible hosts.

Another hypothesis linking resistance to fitness is the inbreeding-infection hypothesis [11], [19], [20]. Inbreeding causes a decrease in heterozygosity due to mating with relatives and is associated with a reduction in fitness, referred to as inbreeding depression [21]. This fitness reduction is expected both in the absence and in the presence of parasites. Several studies reported that inbreeding might also elevate the susceptibility to diseases [19], [20], [22] and thus suggested a correlation between susceptibility and low fitness due to inbreeding. Under this hypothesis, genotypes are both infected and also generally less fit due to inbreeding. It is possible that the negative effects of inbreeding are even more pronounced in the presence of parasites: inbred animals could over-proportionally suffer from parasites [19]. However, this form of interaction between parasitism and inbreeding is not always observed [23].

Both the cost of resistance hypothesis and the inbreeding-infection hypothesis assume that resistance has a genetic basis. Under that assumption, the naturally infected genotypes are infected due to their lack of genetic resistance against parasites and not only due to epidemiology. Here, we first test the assumption of a genetic contribution to resistance in natural populations. We then test the two afore mentioned hypotheses. Testing the cost-of-resistance and the inbreeding-infection hypotheses requires the curing of naturally infected genotypes, which is possible in our system. After curing, the relative fitness of formerly infected, susceptible genotypes can be compared with naturally uninfected and possibly resistant genotypes. The direction of the correlation between resistance and fitness can be determined both in the absence or in the presence of parasites. The two hypotheses make different predictions. The cost-of-resistance hypothesis predicts that the relative fitness of naturally uninfected (and possibly resistant) animals should be lower compared to cured but formerly infected (and thus susceptible) animals in the absence of parasites. In contrast, the inbreeding-infection hypothesis predicts that formerly infected genotypes are those that are also more inbreed and thus should have a lower fitness compared to the naturally uninfected genotypes. When the negative effects of inbreeding are more pronounced in the presence of parasites, inbred animals should even over-proportionally suffer from parasites.

We tested an association of a host's natural infection status and its genotype specific fitness in two experiments (thereafter referred to as infection history experiments). We compared the competitive abilities of naturally uninfected and naturally infected but subsequently cured *Daphnia magna.* The parasite of interest was the microsporidium *Octosporea bayeri*. This parasite has a host genotype specific infectivity and virulence [23], [24]. We measured host fitness in competition experiments that took place both in the presence and absence of sympatric parasite isolates. By that, we could test for an association between the natural infection status of a host and other fitness aspects such as a cost of resistance or a higher inbreeding depression in naturally infected animals. A strength of our study is the use of replicated host genotypes that were collected in 22 natural populations and differed naturally in their infection status. We then assayed their fitness in controlled experiments. We could standardize host age, exclude sex-specific effects and–most importantly–cure hosts from infections while keeping their genotype constant by using only asexually reproducing female *D. magna*.

## Materials and Methods

### The study system

The freshwater crustacean *Daphnia magna* Straus, 1820 (Crustacea: Cladocera) is widely distributed along the coast of the Baltic Sea, inhabiting fresh water rock pools [25]–[27]. These pools are spatially separated and genetic differentiation between the rock pool populations is strong [28]. Periods of asexual reproduction during summer are intermitted by sexual reproduction when resting eggs are produced. The resting eggs allow over-wintering and also serve as dispersal stages [27]. Rock pool *D. magna* populations often harbour a wide spectrum of parasites [1], [29], [30]. The most common parasite in our study area is the microsporidium *Octosporea bayeri* Jírovec, 1936, which can be found in about 45% of all *D. magna* populations [30]. It is vertically as well as horizontally transmitted and reduces host fecundity and survival [24]. *Octosporea bayeri* is specific to *D. magna*. Local adaptation of the parasite occurs on a population level and the locally adapted parasite harms sympatric hosts more than allopatric hosts [31]. This promotes immigration of the allopatric immigrants [31]. Only resting eggs survive during winter and in spring all hatched *Daphnia* are genetically unique recombinants. Spring hatchlings may be *O. bayeri* infected or not. Vertical transmission of *O. bayeri* through the resting egg is highly efficient and infected spring hatchlings descend from infected mothers [24]. The uninfected hatchlings descend either from an uninfected mother or from an infected mother, but lost the parasite during the resting egg stage. The likelihood of vertical transmission through sexual eggs depends on host genotypes and is lower in outcrossed *Daphnia* relative to inbred *Daphnia* [32]. Outcrossing is also beneficial with respect to general competitive abilities, as in this metapopulation a high inbreeding depression was found [33]. No totally resistant genotypes have been found yet, and all *Daphnia* may encounter spores from the sediments or from dead infected hosts and acquire a horizontal infection during the summer season.

### Experimental set-up of the infection experiment

This experiment tested whether the natural infection status correlates with the susceptibility to infections under controlled laboratory conditions. In spring 2003, two female *D. magna* per population were collected from natural populations. All genotypes were kept individually in 100 ml artificial medium [34] at room temperature with a dark/light cycle of 10/14 hours and were fed ad libitum with the green alga *Scenedesmus obliquus*. We checked each genotype for infections of the microsporidium *O. bayeri*. In six populations, one genotype was naturally uninfected and the other infected. All genotypes were treated with the antibiotic fumagillin to cure infections [35]. The success of curing was confirmed afterwards in all genotypes. We then homogenized additional infected individuals from these six populations to prepare spore solutions and inoculated 2- to 3-day-old second-generation offspring of all genotypes with 50'000 sympatric spores per animal [36]. Per genotype, twelve animals were inoculated, kept in pairs in 2.5 ml of artificial medium for five days and fed daily 3×10^5^ algae cells. Animals were transferred to 100 ml medium after five days. Thereafter, they were transferred to fresh medium every third day and fed with 2×10^6^ algae cells per day. After 12 to 16 days they were checked for infections.

### Experimental set-up of the infection history experiments

We experimentally tested if there are fitness differences between naturally uninfected and cured but formerly infected host genotypes. Genotypes from different populations differ strongly in their relative fitness [31], [33]. Therefore a pairwise within-population comparison was used with 22 genotype pairs, each from a different population near Tvärminne Zoological Station, southwest Finland (59°50′N, 23°15′E). Fitness was measured relative to a tester genotype and for eight populations additionally in direct competition within the genotype pair. Relative fitness was measured in population competition experiments both in the presence and in the absence of sympatric isolates of *O. bayeri*. The experiments were performed during the asexual phase of *D. magna.*

In May 2004 we collected *D. magna* females hatched from over-wintering eggs from 22 natural rock pool populations on 12 islands near Tvärminne Zoological Station. As all over-wintering eggs (ephippia) are the result of sexual reproduction, each hatchling is a unique genotype ( = clone). We collected all *Daphnia* shortly after their hatching at a time when they did not yet have started to reproduce. Thereby we were sure that each of them was genetically different [37]. All populations were at least two years old and showed some polymorphism at allozyme loci. However, polymorphism at the allozyme loci is generally rather small in that metapopulation and allozymes do not allow to identify each genotype separately [28]. All populations were known to be infected with *O. bayeri* in the year previous to our studies. Spores of the parasite can be easily seen when an infected *Daphnia* is dissected and investigated with phase-contrast microscopy (400-fold magnification). From each population, we randomly collected one infected and one uninfected female and established clonal isofemale lines ( = asexually reproducing genotypes). We then typed them for their allozyme genotypes at five different loci. These five loci were aspartate amino transferase (*Aat*, enzyme commission number EC 2.6.1.1), fumarate hydratase (*Fum*, EC 4.2.1.2), glucose-6-phosphate isomerase (*Gpi*, EC 5.3.1.9), phosphoglucomutase (*Pgm*, EC 5.4.2.2), and mannose-6-phosphate isomerase (*Mpi*, EC 5.3.1.8) [38]. All genotypes were kept individually in 100 ml artificial medium [34] at room temperature with a dark/light cycle of 10/14 hours and were fed *ad libitum* with the green alga *Scenedesmus obliquus*. To cure the 22 infected genotypes from the *O. bayeri* infections, we treated these genotypes as well as the 22 uninfected genotypes with the antibiotic fumagillin [35]. The success of curing was confirmed afterwards in all genotypes. To avoid maternal effects due to the former infection or the curing procedure, the experiment was started with second-generation offspring of all genotypes. From each genotype about 60 animals were kept in two 250 ml jars, and fed daily with 100 million algae. In parallel, mass cultures of a single uninfected genotype starting from one single female were established. This genotype had been collected in 2003 and kept in the laboratory in its asexual phase ever since then. This genotype differed in at least one of the five allozyme markers to each of the 44 other genotypes and was assigned to be the tester genotype for the competition experiment.

From 28 June to 2 July 2004 the experiment was started. The animals were released into plastic buckets (volume 6 L) containing water filtered through a 20-µm filter from a rock pool free of *D. magna* and free of parasites. The pool from which we took the water was representative for pools found in that metapopulation, though its water quality tends to be in the suboptimal range for *D. magna* [39]. Per 6 liter of filtered pool water, 250 ml sea-water was added to increase salinity, calcium concentration and nutrients. Thereby the water quality was improved for *Daphnia*. However, we did not try to make the water quality optimal, as under optimal conditions a cost of resistance might be obscured [18]. 20 animals of each of the 44 genotypes were released in two buckets respectively, giving in total 88 replicates. Into all of these buckets also 20 animals of the tester genotype were added. For the eight pairs where the two genotypes of a pair could be distinguished with allozyme markers, 20 animals of each genotype were released together in four (for three populations only two) additional buckets respectively. Into half of all the replicates the parasite *O. bayeri* was added, to measure fitness effects in the presence of parasites, while the other half of the replicates stayed free of parasites. The parasite was added to the buckets by placing 30 dead infected *D. magna* females (freshly killed with CO_2_) to each of these replicates. We used sympatric parasites, collected from the 22 corresponding rock pool populations. In five rock pools there were not enough *D. magna* to obtain 30 parasitized animals. In these cases allopatric parasites were used additionally. Parasite spores were passively released from the decaying cadavers of these dead infected *Daphnia*, allowing the infection to spread naturally in the experimental populations. Thirty dead females from parasite free laboratory cultures were added to all parasite free replicates. Also the tester genotype could get infected and its competitive ability may vary in the presence or absence of parasites. Therefore, relative fitness differences between the two treatments ( = parasite treatment effect) do not only incorporate a parasite's effect, but also a tester genotype reaction to the parasite. The two components cannot be separated with our design.

The experiment ran until mid of August 2004, which is equivalent to about five to six asexual *Daphnia* generations. Then, from each replicate a random sample was collected. These animals were genotyped to estimate the frequencies of the two genotypes per replicate (on average 69 animals were genotyped per replicate). Additionally, 20 animals per replicate were homogenized and screened for parasite spores to check the success of the parasite treatment.

### Fitness measurement and analysis

We estimated the relative fitness *w* of a genotype relative to the tester (e. g. A to B) or fitness of naturally uninfected genotypes relative to cured genotypes using the formula ln(*w*)*t = ln(A_t_/B_t_)−ln(A_0_/B_0_) [21], where A_t_, A_0_, B_t_, and B_0_ are the frequencies of the two genotypes A and B at time *t* (measured in days) and time 0. As the experiments took place during asexual reproduction of *D. magna*, only clonal competition occurred and changes in allele frequencies are equal to changes in genotype frequencies. Therefore, ln(*w*) is a comprehensive measurement of relative fitness [21] and was used in the statistical analysis as dependent variable. We did a pairwise comparison between naturally uninfected and cured genotypes (Wilcoxon signed rank test). One replicate was lost in the parasite treatment, as the experimental population went extinct due to unknown reasons. This reduced the number of populations from 22 to 21 in several comparisons. In ten replicates with parasite exposure, the infections did not successfully establish and thus did not allow a pairwise analysis anymore. Therefore we additionally fitted two generalized linear models using both the planned treatments (i. e. exposed to parasites) and the effective treatments (i. e. parasite establishment and spread was observed) as explanatory variables. In the generalized linear models we used genotype frequencies and quasibinomial error structure instead of relative fitness [40]. In all comparisons the biological relevant unit of replication was population. Statistical analyses were performed with R [41].

## Results

### Infection experiment

**Figure 1:**
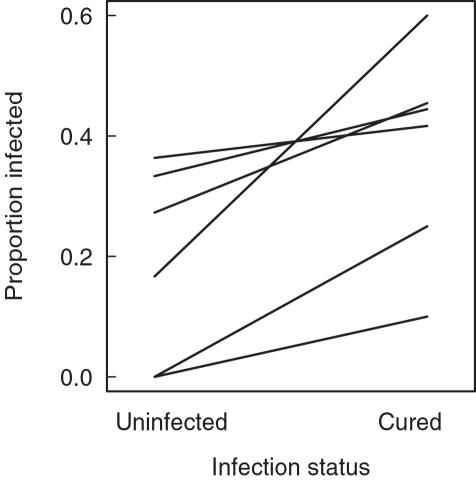
Susceptibility of naturally uninfected and cured *Daphnia* to infection with the parasite *O. bayeri.* We experimentally compared the susceptibility to infection of naturally uninfected *D. magna* and cured but formerly infected *D. magna* (infection status). Each line is the mean of a genotype pair from a different population. Within a population, cured but formerly infected genotypes had a significantly higher likelihood to become infected than naturally uninfected genotypes.

The reasoning of our study hinges on the assumption that the natural infection status is an indicator of resistance. We tested this assumption with pairs of clones from six populations. Within populations, the likelihood to become infected with sympatric parasites is significantly higher for cured but formerly infected genotypes than for naturally uninfected genotypes (paired Wilcoxon signed rank test, *V* = 0, *p* = 0.03, n = 6 pairs; Fig. 1). Mortality during the infection assay did not differ between naturally uninfected and cured genotypes (paired Wilcoxon signed rank test, *V* = 5, *p* = 0.59, n = 6 pairs).

### Infection history experiments

**Figure 2:**
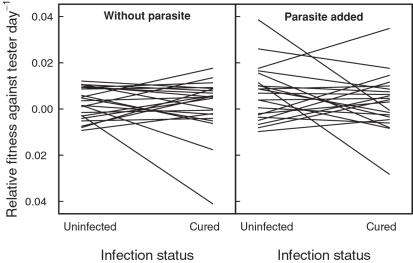
Relative fitness of naturally uninfected and cured *Daphnia* genotypes. Relative fitness within genotype pairs from 22 populations in the absence (left panel) or in the presence of a sympatric parasite (right panel). Within each pair, one genotype was naturally uninfected and the other cured but formerly infected. There was no significant effect of the infection status on a genotypes' competitive ability. The genotype pairs of each population are connected with a line. Fitness was measured for each of the 44 genotypes individually in a competition experiment against a common tester genotype.

**Figure 3 pone-0001280-g003:**
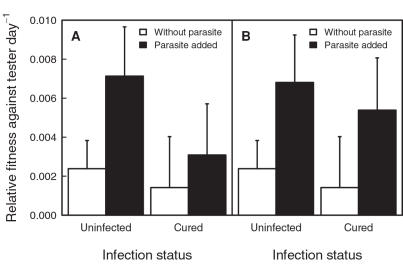
Relative fitness of naturally uninfected and cured *Daphnia* genotypes. Relative fitness within genotype pairs from 22 populations in the absence or in the presence of a sympatric parasite. These are the same data as in Fig. 2, but now mean values (±SE) of relative fitness are given. We furthermore distinguished between the planned treatments where all replicates were analysed (A) and the effective treatments (B). In the effective treatments we excluded replicates in which a successful establishment of the parasite could not be verified.

We did not find a fitness difference between naturally uninfected and cured but formerly infected genotypes both in the absence (paired Wilcoxon signed rank, *V* = 128, *p* = 0.98, n = 22 pairs; Fig. 2a) or in the presence of a sympatric parasite (paired Wilcoxon signed rank test, *V* = 134, *p* = 0.54, n = 21 pairs; Fig. 2b). A generalized linear model with quasibinomial error distribution of genotype frequency as the dependent variable and the explanatory variables infection status, population of origin and parasite treatment was consistent with this analysis: infection status *F*_1,85_ = 1.3, *p* = 0.30; population of origin *F*_21,63_ = 2.8, *p* = 0.001 and parasite treatment *F*_1,84_ = 2.5, *p* = 0.12 (Fig. 3a). The parasite did not successfully establish in 10 out of 43 parasite treatment replicates. Excluding these replicates did not change the results qualitatively: infection status *F*_1,75_ = 0.36, *p* = 0.55; population of origin *F*_21,53_ = 2.8, *p* = 0.001 and effective parasite treatment *F*_1,74_ = 4.6, *p* = 0.04 (Fig. 3b). No replicates of the parasite-free treatment became infected. The mean relative fitness of all genotypes was higher in the presence of parasites than in the absence of parasites. Apparently, the tester genotype was generally a worse competitor in the presence of the parasite.

**Figure 4:**
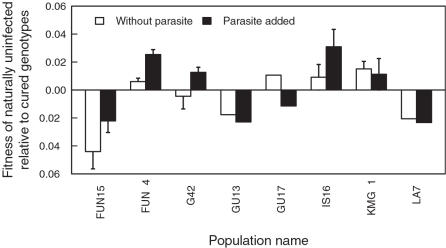
Fitness of naturally uninfected relative to cured *Daphnia* genotypes. Relative fitness difference (mean±SE) per day within genotype pairs from eight populations. In each pair, one genotype was naturally uninfected while the other was cured but formerly infected. Relative fitness was measured in direct competition. There was no effect of the infection status on competitive abilities, as the overall mean was not significantly different from zero. All genotypes were uninfected at the onset of the experiment and in half of the replicates we added sympatric parasites (black bars).

Within pairs from eight populations, genotypes differed by chance in their genetic markers and could therefore be used to measure competitive abilities in direct competition between these genotypes. In this case, fitness differentials were calculated in the perspective of the naturally uninfected genotype relative to the cured genotype. In accordance to the previous experiment, there was no effect of the infection status on relative fitness (generalized linear model with quasibinomial error structure, overall mean estimate = −0.13, *t* = −1.1, *p* = 0.3, population of origin *F*_1,7_ = 8.4, *p* = 0.0002 and parasite treatment *F*_1,24_ = 2.3, *p* = 0.11 (Fig. 4).

**Figure 5:**
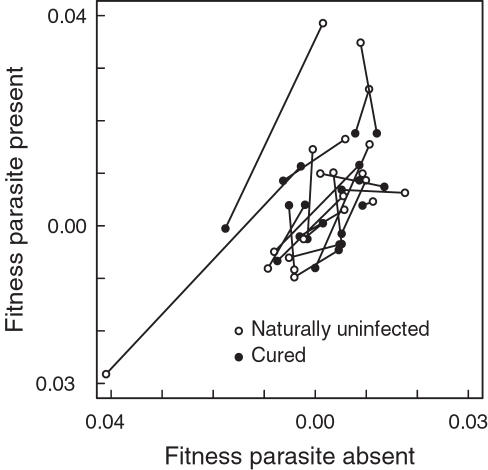
Relative fitness of *Daphnia* genotypes in the presence and in the absence of parasites. Each dot represents the relative fitness of a genotype in the presence of parasites (y-axis) compared with its fitness in the absence of parasites (x-axis). In total, 42 genotypes from 21 populations have been tested. Open dots represent naturally uninfected genotypes, while filled dots represent cured but formerly infected genotypes. We connected the dots of the two genotypes from each population with a line. 16 out of these 21 lines have a positive slope. Thus, within a pair of genotypes, the one that has a high fitness in the absence of parasites has also a high fitness in the presence of parasites, and vice versa (p = 0.02). Within each pair, one genotype was naturally uninfected and the other cured but formerly infected (open and filled dots). However, there was no correlation of the natural infection status with the genotype's relative fitness.

Finally, we compared the relative fitness of *Daphnia* genotypes in the presence and in the absence of parasites. We again used the same fitness values as above that were estimated in competition against a common tester genotype (Fig. 2 & 3). For this analysis we included in total 42 genotypes from 21 populations (the genotypes of one population were excluded, as in one replicate all animals went extinct). We first plotted the fitness of each genotype in the presence of parasites relative to its fitness in the absence of parasites (one dot for each of the 42 genotypes; in Fig. 5). Open dots represent the 21 naturally uninfected genotypes, while filled dots represent the 21 cured but formerly infected genotypes. We connected the values of the two genotypes from each population with a line. 16 out of these 21 lines have a positive slope. Thus, when comparing two genotypes, the one that has the higher fitness in the absence of parasites has on average also the higher fitness in the presence of parasites, and vice versa (Wilcoxon test, *V* = 181, *p* = 0.02; Fig. 5). A negative slope would indicate a trade off when a genotype is either only good in the presence or in the absence of parasites. This was not the case and therefore the results do not support a cost of resistance. The positive relationship we found is in accordance with different levels of inbreeding within populations. In that scenario, some genotypes are generally superior (irrespective of the presence or absence of parasites), while others may be inferior. The latter might be more inbred. Within each pair, one genotype was naturally uninfected and the other one was cured. However, as already shown above, the natural infection status did not correlate with the genotype's relative fitness, which is contrary to the inbreeding-infection hypothesis.

### The use of a tester genotype

**Figure 6:**
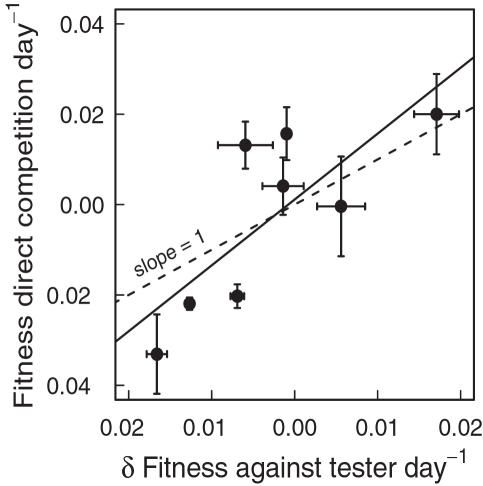
Fitness estimates from direct competition compared with estimates from competition against a common tester genotype. Correlation of mean (±SE) fitness estimates from eight genotype pairs estimated with two different approaches. The first approach used relative fitness of each genotype against a common tester and the difference between naturally uninfected and cured genotypes is calculated (x-axis). The second approach used direct competition within each genotype pair, and fitness of naturally uninfected relative to cured genotypes is given (y-axis). There is a significant positive correlation between these two estimates. Each genotype pair was from a different population. For comparison, we show the slope of one (dashed line), which would be a perfect fit of the two approaches.

Our experiments were mainly based on fitness measurements relative to one tester genotype (Fig. 2. & 3). We corroborated the conclusions of this approach by additionally using direct fitness estimates between genotypes from eight populations (Fig. 4). The results of the two approaches were consistent when comparing the natural infection status and the genotypes' relative fitness (see above). This consistency of the two approaches was also seen when directly comparing the fitness estimates: the fitness estimates against a tester genotype correlated significantly with the fitness estimates from direct competition between the genotypes (linear model; y = 1.46×+0.001; *R* ^2^ = 0.61, *p* = 0.02, n = 8 pairs of genotypes, Fig. 6).

## Discussion

We compared the competitive abilities of naturally uninfected and cured but formerly infected *D. magna* from 22 natural populations. By this, we tested for a correlation between the fitness of a genotype and its natural infection status within populations. All *Daphnia* were cloned and treated against parasites before the experiments. The natural infection status (naturally uninfected vs. cured) did not correlate with a genotype's competitive success, neither in competition against a tester genotype (Fig. 2 & 3) nor in direct competition between the genotypes (Fig. 4). Furthermore, the presence or the absence of a sympatric parasite during the experimental trials did not change the competitive abilities (Fig. 5). Thus, we do not have an indication that other fitness components are related with the natural infection status of *D. magna*. There is no indication for a cost of resistance in our experiment. Likewise, there is no evidence that naturally infected genotypes are generally poor competitors as predicted by the inbreeding-infection hypothesis.

When postulating a correlation between a genotype's natural infection status and its fitness, a genetic basis of susceptibility and thus being infected is assumed. Indeed, cured but formerly infected genotypes had a higher likelihood to become infected compared with naturally uninfected genotypes (Fig. 1). This shows that susceptibility to infection has a genetic basis within host populations and that this genetic effect influences the likelihood to be infected under natural conditions. Such an increased susceptibility of formerly infected genotypes to infections has also been shown in the *Daphnia magna-Pasteuria ramosa* system [5], [6].

In a cost of resistance scenario, a trade-off is assumed between resistance and other fitness components [18], [42]. However, neither in our system nor in the *D. magna-P. ramosa* system was evidence for a cost of resistance found [5], [6], [10]. Naturally uninfected *Daphnia* did not have a lower fitness compared with cured but formerly infected genotypes in the absence of parasites (Fig. 2–4). Some genotypes were better competitors irrespective of the presence or the absence of parasites and irrespective of their natural infection status (Fig. 5). The absence of a cost of resistance is somewhat surprising. The parasite *O. bayeri* decreases host fitness significantly [24], [36] and is highly abundant in the studied metapopulation [30]. Also *P. ramosa* is highly virulent and common in its host populations [5], [6], [43]. Therefore, even rather costly mechanisms should be selected for if they reduce susceptibility to disease. A possible explanation could be trade-offs in resistance against different parasites (and costs paid for the resistance). In our experiments we did not test for a cost that comes in the currency of resistance to other parasite species or to other than sympatric *O. bayeri* isolates [15], [34]. Such a cost may be sufficient to maintain variation for resistance and could theoretically explain our results. We restricted ourselves to *Octosporea bayeri*, as it is by far the most common parasite in this metapopulation and all other parasites are rarer [31]. Thus *Daphnia* might only profit from a scenario that includes other parasites in a limited number of cases. However, for future studies it would be worth to include different parasites.

Little et al. [10] compared in the laboratory generally resistant genotypes with susceptible genotypes. It may be argued that the resistance seen in the laboratory did not reflect the natural situation because the outcome of host-parasite interactions may depend on environmental conditions such as temperature [44]. Here we used the natural infection status as explanatory variable and performed our experiments under natural outdoor conditions, but still did not find a cost of resistance. This suggests that such a relation may not be important in our system. An alternative, which we cannot rule out, is that the costs are seen only for fitness components not assessed in our experiments. Zbinden et al. [45] provide evidence that resistant *D. magna* genotype showed a reduced clonal growth rate, but only under stress free conditions (i. e. without interspecific competition). However, this might not be a frequent situation, as both inter- and intraspecific competition is common in this system [46].

Inbreeding is another fitness aspect to which a genotype's infection status might be related [11], [19], [20]. Inbreeding generally causes a decrease in fitness [21] and is common in the herein studied metapopulation due to frequent bottlenecks during colonization of empty rock pools [33], [47], [48]. However, it is unclear if inbreeding influences the likelihood to be infected within a natural population. The inbreeding-infection hypothesis postulates that cured but formerly infected genotypes should have a lower fitness compared to naturally uninfected genotypes. The sign of this correlation did not change in the absence compared to the presence of parasites in the competition trials, which is contrary to the predictions from the cost of resistance scenario. This arises, because inbred animals are expected to be consistently worse competitors [23], [49]. Parasites may intensify the negative effects of inbreeding, but are not expected to change the sign of the association.

In our experiments we found a significant population effect (Fig. 2 and 3), possibly due to different degrees of inbreeding and inbreeding depression between populations. Furthermore, within populations some genotypes were generally better competitors than others irrespective of the presence or the absence of parasites (Fig. 5). These might be genotypes that suffered less from inbreeding depression. However, their relative fitness was not related to their natural infection status, and within a population cured but formerly infected animals were not generally worse competitors as suggested by the inbreeding infection hypothesis (Fig. 2–4). Thus, we conclude that different degrees of inbreeding within populations did not explain variation in resistance to *O. bayeri*.

The natural infection status can be important in a metapopulation context. In a previous study [31] we showed that uninfected migrants are more successful than infected migrants. The current study emphasizes the importance for migrants to be free of parasites: there is no general fitness difference between susceptible and resistant genotypes, and thus being actually infected is a supplementary negative fitness component. Migrants entering populations with or without parasites would do best in any case being uninfected and resistant, as resistant immigrants will not have a disadvantage in uninfected resident populations.

In all experiments we used competitive ability as an integrative measurement of the overall fitness rather than individual fitness components such as carrying capacity or reproductive success [50]–[52]. Fitness values are implicitly taken as additive and being ordered, meaning that if fitness of genotype A>fitness of genotype B, and B>C, then A>C [21]. We did not test directly this assumption, but our two approaches, namely a comparison of fitness measurements from direct competition between genotypes and second using fitness differences from competition against tester genotypes gave consistent results (Fig. 6). Thus, we assume that our competition trials give meaningful fitness estimates.

In conclusion, we did not find a correlation between the natural infection status (naturally uninfected vs. cured) of a genotype and its competitive success. This was the case both in the presence as well as in the absence of sympatric parasites during the competition experiments. Therefore, our data do not support the inbreeding-infection hypothesis. They also do not support a cost of resistance, however ignoring potential costs caused by other parasite strains or parasite species. We suggest as a possible explanation for our results that resistance genes might segregate largely independently of other fitness associated genes in this system.

## References

[1] Ebert D (2005). Ecology, Epidemiology, and Evolution of Parasitism in *Daphnia*..

[2] Poulin R (1998). Evolutionary Ecology of Parasites: from Individuals to Communities..

[3] Hudson P, Greenman J (1998). Competition mediated by parasites: biological and theoretical progress.. Trends in Ecology & Evolution.

[4] Minchella DJ, Scott ME (1991). Parasitism-a cryptic determinant of animal community structure.. Trends in Ecology and Evolution.

[5] Duncan AB, Mitchell SE, Little TJ (2006). Parasite-mediated selection and the role of sex and diapause in *Daphnia*.. Journal of Evolutionary Biology.

[6] Little TJ, Ebert D (2000). The cause of parasitic infection in natural populations of *Daphnia* (Crustacea: Cladocera): the role of host genetics.. Proceedings of the Royal Society of London Series B-Biological Sciences.

[7] Rauch G, Weisser WW (2006). Local and spatial dynamics of a host-parasitoid system in a field experiment.. Basic and Applied Ecology.

[8] Brown MJF, Loosli R, Schmid-Hempel P (2000). Condition-dependent expression of virulence in a trypanosome infecting bumblebees.. Oikos.

[9] Bandi C, Dunn AM, Hurst GDD, Rigaud T (2001). Inherited microorganisms, sex-specific virulence and reproductive parasitism.. Trends in Parasitology.

[10] Little TJ, Carius HJ, Sakwinska O, Ebert D (2002). Competitiveness and life-history characteristics of *Daphnia* with respect to susceptibility to a bacterial pathogen.. Journal of Evolutionary Biology.

[11] Keller LF, Waller DM (2002). Inbreeding effects in wild populations.. Trends in Ecology and Evolution.

[12] Lively CM (1989). Adaptation by a parasitic trematode to local-populations of its snail host.. Evolution.

[13] Nee S (1989). Antagonistic co-evolution and the evolution of genotypic randomization.. Journal of Theoretical Biology.

[14] Sasaki A (2000). Host-parasite coevolution in a multilocus gene-for-gene-system.. Proceeding of the Royal Society B-Biological Sciences.

[15] Carius HJ, Little TJ, Ebert D (2001). Genetic variation in a host-parasite association: potential for coevolution and frequency-dependent selection.. Evolution.

[16] Carton Y, Nappi AJ, Poirie M (2005). Genetics of anti-parasite resistance in invertebrates.. Developmental and Comparative Immunology.

[17] Little TJ, O'Connor B, Colegrave N, Watt K, Read AF (2003). Maternal transfer of strain-specific immunity in an invertebrate.. Current Biology.

[18] Kraaijeveld AR, Godfray CH (1997). Trade-off between parasitoid resistance and larval competitive ability in *Drosophila melanogaster*.. Nature.

[19] Coltman DW, Pilkington JG, Smith JA, Pemberton JM (1999). Parasite-mediated selection against inbred Soay sheep in a free-living island population.. Evolution.

[20] Reid JM, Arcese P, Keller LF (2003). Inbreeding depresses immune response in song sparrows (*Melospiza melodia*): direct and inter-generational effects.. Proceeding of the Royal Society B-Biological Sciences.

[21] Hartl DL, Clark AG (1997). Principles of population genetics..

[22] Luong LT, Heath BD, Polak M (2007). Host inbreeding increases susceptibility to ectoparasitism.. Journal of Evolutionary Biology.

[23] Salathé P, Ebert D (2003). The effects of parasitism and inbreeding on the competitive ability in *Daphnia magna*: evidence for synergistic epistasis.. Journal of Evolutionary Biology.

[24] Vizoso DB, Ebert D (2004). Within-host dynamics of a microsporidium with horizontal and vertical transmission: *Octosporea bayeri* in *Daphnia magna*.. Parasitology.

[25] Pajunen VI, Pajunen I (2003). Long-term dynamics in rock pool *Daphnia* metapopulations.. Ecography.

[26] Bengtsson J (1989). Interspecific competition increases local extinction rate in a metapopulation system.. Nature.

[27] Ranta E (1979). Niche of *Daphnia* species in rockpools.. Archiv für Hydrobiologie.

[28] Haag CR, Riek M, Hottinger JW, Pajunen VI, Ebert D (2005). Genetic diversity and genetic differentiation in *Daphnia* metapopulations with subpopulations of known age.. Genetics.

[29] Bengtsson J, Ebert D (1998). Distribution and impacts of microparasites on *Daphnia* in a rockpool metapopulation.. Oecologia (Berlin).

[30] Ebert D, Hottinger JW, Pajunen VI (2001). Temporal and spatial dynamics of parasites in a *Daphnia* metapopulation: Which factors explain parasite richness?. Ecology.

[31] Altermatt F, Hottinger J, Ebert D (2007). Parasites promote host gene flow in a metapopulation.. Evolutionary Ecology.

[32] Ebert D, Altermatt F, Lass S (2007). A short term benefit for outcrossing in a *Daphnia* metapopulation in relation to parasitism.. Journal of the Royal Society Interface.

[33] Ebert D, Haag C, Kirkpatrick M, Riek M, Hottinger JW (2002). A selective advantage to immigrant genes in a *Daphnia* metapopulation.. Science.

[34] Klüttgen B, Dülmer U, Engels M, Ratte HT (1994). ADaM, an artificial freshwater for the culture of zooplankton.. Water Research.

[35] Zbinden M, Lass S, Refardt D, Hottinger J, Ebert D (2005). *Octosporea bayeri:* fumidil B inhibits vertical transmission in *Daphnia magna*.. Experimental Parasitology.

[36] Vizoso DB, Ebert D (2005). Mixed inoculations of a microsporidian parasite with horizontal and vertical infections.. Oecologia.

[37] Haag CR, Ebert D (2007). Genotypic selection in *Daphnia* populations consisting of inbred sibship.. Journal of Evolutionary Biology.

[38] Hebert PDN, Beaton MJ (1993). Methodologies for allozyme analysis using cellulose acetate electrophoresis (2. edition)..

[39] Pajunen VI, Pajunen I (2007). Habitat characteristics contribution to local occupancy and habitat use in rock pool *Daphnia* metapopulations.. Hydrobiologia.

[40] Crawley MJ (2002). Statistical computing. An introduction to data analysis using S-Plus..

[41] R Development Core Team (2007). R: A language and environment for statistical computing. Version 2.4.0. 2.4.0 ed..

[42] Groeters FR, Tabashnik BE, Finson N, Johnson MW (1994). Fitness costs or resistance to *Bacillus thuringiensis* in the diamondback moth (*Plutella xylostella*).. Evolution.

[43] Jensen KH, Little T, Skorping A, Ebert D (2006). Empirical support for optimal virulence in a castrating pparasite.. Public library of Science Biology.

[44] Mitchell SE, Rogers ES, Little T, Read AF (2005). Host-parasite and genotype-by-environment interactions: Temperature modifies potential for selection by a sterilizing pathogen.. Evolution.

[45] Zbinden M, Haag CR, Ebert D (2008). Rapid host adaptation to a parasite in experimental field populations of *Daphnia magna*..

[46] Bengtsson J (1993). Interspecific competition and determinants of extinction in experimental populations of three rockpool *Daphnia* species.. Oikos.

[47] Haag CR, Hottinger JW, Riek M, Ebert D (2002). Strong inbreeding depression in a *Daphnia* metapopulation.. Evolution.

[48] Haag CR, Riek M, Hottinger JW, Pajunen VI, Ebert D (2006). Founder events as determinants of within-island and among-island genetic structure of *Daphnia* metapopulations.. Heredity.

[49] Haag CR, Sakwinska O, Ebert D (2003). Test of synergistic interaction between infection and inbreding in *Daphnia magna*.. Evolution.

[50] Baganz F, JHayes A, Farguhar R, Butler PR, Gardner DCJ (1998). Quantitative analysis of yeast gene function using competition experiments in continuous culture.. YEAST.

[51] Capaul M, Ebert D (2003). Parasite-mediated selection in experimental *Daphnia magna* populations.. Evolution.

[52] Fellowes MDE, Kraaijeveld AR, Godfray CHJ (1998). Trade-off associated with selection for increased ability to resist parasitoid attack in *Drosophila melanogaster*.. Proceeding of the Royal Society B-Biological Sciences.

